# The Mystery of the Hidden Trace: Emerging Genetic Approaches to Improve Body Fluid Identification

**DOI:** 10.3390/genes17020146

**Published:** 2026-01-28

**Authors:** Dana Macfarlane, Gabriela Roca, Christian Stadler, Sara C. Zapico

**Affiliations:** 1 Department of Chemistry and Environmental Sciences, New Jersey Institute of Technology, Newark, NJ 07102, USA; dm947@njit.edu; 2 SERATEC^®^ Gesellschaft für Biotechnologie mbH, 37079 Göttingen, Germany; gabriela.roca@seratec.com (G.R.); stadler@seratec.com (C.S.); 3 Anthropology Department and Laboratories of Analytical Biology, National Museum of Natural History, Smithsonian Institution, Washington, DC 20560, USA

**Keywords:** body fluid identification, forensic genetics, forensic serology, molecular forensics

## Abstract

Body fluid identification at crime scenes is the first step in the forensic biology workflow, leading to the identification of the perpetrator and/or, in some cases, the victim. Current methods that are regularly used in forensic criminal evidence analysis utilize well-studied properties of each fluid as the foundation of the protocol. Among these approaches, alternative light sources, chemical reactions, lateral flow immunochromatographic tests, and microscopic detection stand out to identify the main body fluids encountered at crime scenes: blood, semen, and saliva. However, these often come with limits for specificity and sensitivity. There is also difficulty with fluid mixtures, environmental degradation, and destruction of the sample by the method used. Other fluids, like vaginal fluid and fecal matter, lack standardized protocols and require innovative ideas for accurate analysis without compromising the sample. Emerging technologies based on molecular methods have been the focus of body fluid research, with emphasis on topics such as mRNA, microRNA, epigenetics, and microbial analysis. Additional information alongside the determination of fluid origin could be an advantage from new molecular techniques, such as the identification of donors from SNP analysis, if regular STR analysis is not possible. Validation studies and the integration of such research have the potential to expand and enhance the laboratory practices of forensic science. This article will provide an overview of the current methods applied in the crime lab for body fluid identification before exploring active research in this field, pointing out the potential of these techniques for application in forensic cases to overcome present issues and expand the variety of body fluids identified.

## 1. Introduction

The identification of body fluid is an important part of forensic science for crime scene reconstruction and DNA evidence found can be further contextualized by the establishment of the origin of the DNA. Body fluid identification is normally performed before DNA analysis and can help guide which method will provide the clearest results, such as a differential extraction for semen stains [1,2]. Current non-destructive identification methods have the advantage of preserving evidence, because the analyzed portion of the sample can still be used for further identification, but may also be less specific and provide minimal information [3,4,5]. The classic use of an alternative light source (ALS) for the identification of body fluid stains remains such an easy and efficient method that it has been used by laymen for hygiene inspections in hotel suites, for example, though the false positives of ALS have been well documented [6,7]. Immunochromatographic assays are popular for their ease of use and rapid detection, even at crime scenes, though false positives can still occur [8,9]. Additionally, because there are a variety of commercially available tests to choose from, assessing different proteins, not all labs will use the same brands [10]. Microscopic techniques have historically been reliable, but require time, materials, and expertise [11].

Certain fluids have few or no standard identification techniques due to their unique (or rather, not unique) nature. Vaginal fluid, for example, has been a known false positive for semen acid phosphatase tests [12]. Much current research focuses on molecular protocols, the difficulty to pin down fluids, and the potential for multiplexing fluid identification [13,14]. This review will briefly discuss current body fluid identification methods used in forensic labs for blood, semen, saliva, and other, less common fluids. Next, recently published research on new methods will be described with emphasis on molecular techniques and fluids without standard procedures.

## 2. Current Methods

**A.**  **BLOOD**

Blood often appears at crime scenes as small, dry stains. Due to the nature of crimes, it is an extremely common body fluid encountered in forensic analysis [15]. Historically, blood has been identified using phenolphthalein chemical tests (also known as Kastle–Meyer) and, less commonly, leuco-malachite, both of which take advantage of hemoglobin’s peroxidase-like activity and rely on a color change to indicate a positive sample [15,16]. False positives, along with the fading out of direct chemical tests and influx of more efficient assays in forensic laboratories, have caused the use of these to decline [17]. Luminol spray has been an efficient method for viewing invisible blood stains with chemiluminescence from oxidation of the luminol molecule. The spray has been shown to have fewer false negatives than phenolphthalein and leuco-malachite, as well as little to no impact on further molecular processing [3,18,19]. False positives can still occur, such as with various vegetables and paints [20]. Bluestar^®^ is a specific luminol-based spray noted for the stronger chemoluminescence and sensitivity than standard luminol [21]. Hemastix^®^ test strips (Siemens Healthcare, Malvern, PA, USA) also use the peroxidase-like activity of hemoglobin to create a color change with high sensitivity and little impact on downstream DNA analysis, but lack specificity [22,23], as false positives have been seen with vegetables, cleaning products, and acids [17].

For more confirmatory tests for blood, one may use the Takayama assay, which forms hemochromagen crystals under the microscope, though this is not specific to humans and can be time consuming [11]. Immunochromatographic assays have been developed that are rapid and human-blood specific. “Rapid” typically means within 5–10 min. ABAcard^®^ HemaTrace^®^ (Abacus Diagnostics, West Hills, CA, USA) was the first commercially available immunochromatographic test, starting in 2003, which focuses on human hemoglobin and shows high sensitivity [24]. This increased sensitivity is optimal in diluted samples, as false positive results could be encountered with other body fluids and primate blood. The high-dose hook effect could occur in raw samples, being solved by the dilution of the fluid [25]. Another sensitive cartridge test for human hemoglobin is the SERATEC^®^ HemDirect Hemoglobin (SERATEC^®^, Göttingen, Germany) test, which shows less false positive occurrence than HemaTrace^®^ and detects aged blood on a variety of fabrics [25,26]. The RSID^TM^-Blood assay (Independent Forensics, Lombard, IL, USA) is the most specific for human blood, detecting human glycophorin A, but this specificity may reduce sensitivity to more diluted samples [25,27]. Overall, there are a variety of validated blood tests for forensic laboratories to work with depending on the necessities of the crime scene evidence.

**B.**  **SALIVA**

Saliva stains may appear at crime scenes and provide additional opportunities for DNA analysis, especially in those of a sexual nature [28]. There are less validated tests for saliva identification than blood or semen. Saliva, unlike blood, is clear and may dry clear, making identification of a stain difficult. ALSs are commonly used for viewing invisible stains, although saliva stains may lead to false negatives from their weak fluorescence and there are several false positives, including common cosmetics [4,6,29,30]. Other presumptive tests detect activity of α-amylase, a digestive enzyme found in saliva. The Phadebas^®^ Forensic Press Test, also referred to as Phadebas^®^ paper (Phadebas Forensics, Cambridge, MA, USA), and the SERATEC^®^ Amylase Paper, utilize water-insoluble starch polymers bonded to a blue dye to visualize saliva stains [5]. This starch–dye complex has been around for decades and is highly sensitive to amylase, which results in false positives from other body fluids, plants, and cleaning solutions [5,31]. Another commercially available colorimetric test is the SALIgAE^®^ from Abacus Diagnostics, in which a solution turns from colorless to yellow with trace amounts of saliva [32]. Modern forensic laboratories tend to prefer lateral flow immunochromatographic assays of all fluids for their reliability and ease of use. RSID^TM^-Saliva specifically tests for the presence of human salivary α- amylase and has been shown to have limited cross-reactivity with other body fluids [33]. The sensitivity of this assay has also been demonstrated to be significantly greater than Phadebas^®^ paper [28]. The SERATEC^®^ saliva tests detect the presence of α-amylase, with positive results appearing up to 180 days after deposition, though this may be impacted by weather and fabric [34,35]. Additionally, DNA extraction from these tests has been shown to be possible, allowing for extended use of limited evidence samples [36]. While there are several options available for saliva identification, there are conflicting opinions on whether any can stand alone without confirmation with DNA analysis due to cross-reaction with other body fluids [37].

**C.**  **SEMEN**

The discovery and analysis of semen stains is often essential evidence for sexual assault cases and can provide a clearer picture of the crime. At a crime scene and/or on evidence brought to the lab, a non-destructive, alternative light source can help to visualize stains that may be invisible to the naked eye. Semen will dry clear on fabrics or other surfaces but will have significant brightness with ALSs. It has been shown that this brightness can be seen for up to two months after deposition [30]. As previously mentioned, ALSs are not specific to one fluid and can therefore result in false positives [6]. STK^®^ Sperm Tracker^TM^ (AXO Science, Villeurbanne, France) has been developed as an alternative to ALSs that is more specific to seminal fluid. The STK spray^®^ is deposited onto evidence and stains are revealed with a UV light. This test is shown to be strongly specific for seminal fluid [38]. There has been no known interference with DNA analysis or microscopic techniques from sources treated with this spray [39].

Acid phosphatase (AP) is the enzyme detected by Sperm Tracker^TM^ and has been long discovered to be predominantly found in seminal fluid [40]. Testing for AP can be performed by traditional laboratory means by making an AP reagent to detect a color change [23]. However, false positives from oral and vaginal samples can occur [2]. Prostate-specific antigen (PSA), also called p30, is a protein made by the prostate gland discovered to be specifically found in seminal fluid [41]. PSA can also be found in female vaginal fluid up to 24 h post-coitus and in male urine, but is generally shown to be more specific and sensitive than AP [12,42,43]. Investigation into fabric on body fluid identification showed that all components of seminal fluid may not soak through the fabric, thus leading to a false negative for p30 if testing is performed on the underside of a stain [23]. Collection on the top side of a stain is not altered by fabric type [26].

Commercially available immunochromatographic tests have been developed for PSA testing of forensic samples. The ABAcard^®^ p30 test is an option for forensic laboratories for semen stain screening. Diluted fresh and frozen semen samples provide a true positive result with this test, as well as post-vasectomy semen and post-coital vaginal samples. Fluid mixtures with diluted semen may be below the detection limit; however, this depends on the concentration of the semen [8]. The SERATEC^®^ PSA Semiquant is another immunochromatographic test for semen stains that has similar results to ABAcard^®^ p30, with slightly increased sensitivity [44]. Seminal fluid was able to be detected by SERATEC^®^ PSA after 14 days of tropical weather exposure [34]. Both the ABAcard^®^ p30 test and SERATEC^®^ PSA Semiquant test were able to detect seminal fluid from a room temperature-stored 30-year-old stain [45]. PSA and residual semen can occur in male urine. In female urine PSA may be present as well, but in much lower levels; however, routine swab/cutting extraction typically dilutes urinary PSA below rapid-test reactivity [9,12,46]. Post-mortem rectal samples can produce high rates of PSA results, especially from male cadavers [47,48]. The RSID^TM^-Semen lateral flow assay is another semen identification test that utilizes semenogelin, a protein produced in the seminal vesicles and present in seminal plasma [48]. Semenogelin protein detection for the verification of semen presence possibly has fewer false positives and negatives compared to p30 tests, though more statistics are needed; semenogelin tests also have lower sensitivity when handling diluted samples. Also, semenogelin has been found in male urine. [12,46,49,50,51].

The most definitive way of confirming semen presence is microscopic sperm observation. Traditional Christmas tree staining aids in microscopic examination by staining the head of the sperm red and the neck and tail green [11]. The SPERM HY-LITER^TM^ kit (Independent Forensics of Illinois, Lombard, IL, USA) increases the efficiency of microscopic technique with fluorescently labeled human sperm-specific antibodies, glowing green when viewed with an FITC-compatible filter. Sperm cells are able to be visually separated from epithelial cells, though all cell types can be seen using a DAPI filter if desired [52]. While this is a clear identifier, seminal fluid stains may have few or no sperm, such as with post-vasectomy males [8]. It may be necessary to perform more than one semen identification test for strong confirmation of the presence of seminal fluid.

**D.**  **OTHER**

Several other body fluids may be present as evidence and require identification. As previously mentioned, ALSs will show fluorescence in a variety of fluids, including urine, tears, and vaginal fluid, as well as numerous food and household products [4,6,29,30,53,54].

Vaginal fluid is also a known false positive for AP testing, and vaginal acid phosphatase can be distinguished from the prostate enzyme using gel electrophoresis [11]. Microscopically, vaginal fluid can be seen by the visual presence of epithelial cells using Dane’s staining method and identification of vaginal microbes. These methods are not commonly used, however, and vaginal fluid remains a difficult sample type to confirm [54].

Menstrual blood can be difficult to distinguish from venous blood. D-dimer is a protein fragment found when the body breaks down blood clots, which may be present in menstruation. The SERATEC^®^ PMB test detects D-dimer and human hemoglobin for possible presence of menstrual blood. False positive tests may result from individuals with deep vein thrombosis, embolisms, infections, pregnancy, and from post-mortem blood samples [45,55]. Additionally, age and weather conditions may degrade a sample, leading to the inability to identify [55,56].

For urine identification, utilization of the p-Dimethylaminocinnamaldehyde (DMAC) assay for urea presence is a common practice. This test may have false positives with vaginal fluid, semen, saliva, and sweat [57]. A colorimetric test for the detection of creatinine, Jaffe’s test, can also be performed for presumptive urine identification. False positives from semen and the hazardousness of the chemicals involved have made the use of this test uncommon [11]. The Tamm–Horsfall Protein, a glycoprotein found in the urine and kidneys, can be tested for using the immunochromatographic assay RSID^TM^-Urine test. Fluid mixtures, however, may cause inhibition, resulting in false negatives [58].

Feces identification would primarily be performed using a combination of microscopic analysis and testing for urobilinoids. The presence of broken-down foodstuffs and specific bacteria, such as Bacteroides uniformis, could indicate fecal matter. This does not distinguish between human and animal fecal matter and may also vary based on individual diet. Chemical testing for urobilinoids, or a form of broken-down heme, could also suggest feces, though again, this does not distinguish between species and could also be found in urine [11].

A summary of the current methods is depicted in Table 1.

## 3. Current Research

While there are several currently used identification assays, scientists continue to work on improvement in the field. Present research focuses on the application of molecular techniques, the simultaneous analysis of multiple body fluids, and the development of assays for fluids with little to no confirmatory testing. These methods have been popular in forensic research, particularly mRNA, which has been the point of investigation for almost three decades [59]. MicroRNAs, epigenetics, and microbial genetics are additional important molecular research areas spanning multiple fluids [60,61]. Several non-destructive methods, including Raman spectroscopy, are also minor points of research [62]. The following section will detail findings from recent publications on these topics in forensics.

**A.**  **mRNA**

Messenger RNA, or mRNA, is transcribed by DNA to become a protein and is regulated by cells to be tissue-specific [14]. Currently, mRNA is used in a variety of clinical research, from vaccines to cancer, with the field of forensics utilizing the same or similar techniques and instrumentation [63]. Analysis of RNA in forensics has been around since the 1980s, first focusing on post-mortem specimens [14]. mRNA has been investigated for body fluid identification purposes due to the specificity and simultaneous DNA amplification that can occur, especially when handling small amounts of a sample [14].

Several established methods in mRNA extraction, amplification, and detection are well recognized in forensic research. Commercial extraction kits are used in-house by many researchers, and it does not appear to have a significant impact on the result depending on which extraction and quantification methods are used [64]. DNA and RNA are both important in forensics, so coextraction and re-extraction are both popular. Coextraction combines multiple steps in one process and can maximize data from a small starting sample but may cause lower relative fluorescence unit (RFU) values [65]. Re-extraction isolates the DNA and RNA in separate workflows and can be especially useful to obtain high yields for the nucleic acid of interest [66]. Not all studies include DNA in their mRNA research and instead focus solely on RNA extraction [67]. In cases which also include microRNA, it is sufficient to use a microRNA extraction kit to collect both types of nucleic acids at once [68].

One popular technique is real-time quantitative reverse transcription polymerase chain reaction (RT-qPCR) for amplification and quantification of a handful of mRNA markers at once using fluorescent probes [67,69,70,71,72,73]. This method has been shown to be sensitive and efficient for body fluid mRNA identification due to requiring no post-PCR processing to acquire read counts, or the total number of times a specific sequence is detected [70,74]. The downsides of RT-qPCR in forensics are the inability to target multiple types of nucleic acids in one tube, such as mRNA and microRNA, as well as no sequencing information if that is of interest [14].

Standard reverse transcription polymerase chain reaction (RT-PCR) is often used in forensic mRNA research and is followed by capillary electrophoresis (CE) or sequencing for marker detection [64,65,68,73,75,76,77,78,79,80,81,82,83,84,85,86,87,88,89]. Capillary electrophoresis utilizes fluorescence and molecule size to distinguish markers and is already used for DNA analysis of short tandem repeats in forensics [90]. More than one type of nucleic acid can be run in the same test and specific single nucleotide polymorphisms (SNPs) located on mRNA genes can be identified, allowing for more information to be procured from the same amount of sample compared to RT-qPCR [66,89]. Sequencing of the amplified mRNA has been investigated for the identification of the body fluid tissue origin in single and mixture stains, as well as donor determination [64,82]. This method provides a great deal of information to be discerned, allowing for numerous SNPs to be recorded at once, but is often the most expensive and time-consuming process [75]. Research into mRNA sequencing has been investigated using Sanger, Illumina Inc., and QNome (Qitan Technology), to name the most common [64,75,82].

Other, less common forms of amplification and detection have been investigated in recent years. One-step endpoint RT-PCR, which combines DNA polymerase and the reverse transcription enzyme in a single tube, has been compared to two-step RT-PCR and found similar results, and does not have advantages in terms of time and/or money [83,88]. Reverse transcription loop-mediated isothermal amplification with CRISPR (RT-LAMP + CRISPR-Cas12a) is a form of fluorescence detection using Cas12a cleavage activity, which has been used in disease diagnostics and therefore its potential in forensics has been researched [91]. Although cheaper compared to RT-qPCR, RT-LAMP is still a two-step process and is also not sensitive enough for forensic use [74]. Real-time reverse transcription recombinase polymerase amplification (RT-RPA) is efficient due to its one-step process; however, only one marker at a time can be tested and it is not very well studied for forensic purposes [69,92].

Generally, the selection of detection methods for mRNA is dependent upon efficiency and the desired information. Forensic mRNA research spans from quick, single-marker testing to multiplexed sequencing. Current research builds upon previously standardized techniques, such as capillary electrophoresis, and branches out into untested methods to further expand the capabilities of forensic mRNA analysis.

Peripheral blood was the first fluid to be investigated for application of mRNAs, resulting in several highly researched markers over the years, including hemoglobin beta (HBB), anion exchange protein (SLC4A1), beta spectrin (SPTB), hemoglobin alpha (HBA), porphobilinogen deaminase (PBGD), and aminolevulinate synthase 2 (ALAS2) [70,83,85,86,93,94]. Other investigated markers for blood include junction adhesion molecule-like (AMICA1), CD93 molecule (CD93), ankyrin-1 protein (ANK1), hemoglobin delta (HBD), and CD3-gamma polypeptide (CD3G) [66,69,73,86,95].

Although there are some conflicting results, several recent studies demonstrate that HBB is detected at smaller dilutions than SLC4A1, ALAS2, and SPTB in blood-only samples [65,76,96]. While HBB withstands environmental conditions compared to ALAS2, fluid mixture may decrease the detectability of HBB [70,73,83]. SLC4A1 was still shown to have sporadic detection at very low dilution and a higher detection rate than ALAS2 [74,85]. Conflicting results suggest that ALAS2 is more specific and equally as sensitive as HBB, however [86]. The RNA expression panel kit used can also impact the amount of reads per marker, leading to different results [97]. Regardless, one study sending mock casework to laboratories found that a majority utilized ALAS2, with HBB being the second most common blood mRNA marker [95].

The predominant saliva mRNA markers researched are statherin (STATH) and histatin 3 (HTN3), which are both associated with the maintenance of oral health [65,66,69,71,74,75,76,79,80,82,83,86,88,92]. Other markers have been investigated, such as mucin 7 (MUC7), follicular dendritic cell-secreted protein (FDSCP), keratin 4 (KRT4), proline-rich protein 4 (PRB4), and proline-rich protein HaeIII subfamily 2 (PRH2) [66,71,74,75,76,79,80,82,83,86,92]. HTN3 and STATH have both been shown to be in virtually all saliva samples analyzed without showing up in other fluids [65,71,80,88]. STATH does, however, appear in nasal secretions, while HTN3 does not [71]. MUC7 has not been able to be detected in all saliva samples and additionally may appear in vaginal samples [14,80]. PRH2 was also found to not be expressed in all saliva samples [71]. FDSCP has lower read counts and general decreased sensitivity than HTN3 [74,83]. Often, HTN3 has higher reads than STATH, though STATH may be more sensitive at increased dilutions [65,76,88]. Not surprisingly, the study that sent mock casework to laboratories found that a majority used HTN3 and STATH [95].

The most used mRNA markers for semen are protamine 1 (PRM1), protamine 2 (PRM2), semenogelin 1 (SEMG1), semenogelin 2 (SEMG2), and transglutaminase 4 (TGM4) [65,66,69,74,75,76,79,80,82,84,86,88,94,95,98]. Other presently investigated markers include NK3 homeobox 1 (NKX3-1), creatine kinase B (CKB), kallikrein-related peptidase 2 (KLK2), kallikrein-related peptidase 3 (KLK3), PRAC1 small nuclear protein (PRAC1), transition protein-1 (TNP1), microseminoprotein beta (MSMB), and sorbitol dehydrogenase (SORD) [65,66,70,74,79,80,82,83,84,86,88,95,98]. As noted before, the semenogelin protein is used in a lateral flow assay for seminal fluid identification [49]. Marker prominence also can vary by person and quality of semen. For example, PRM1, PRM2, and TNP1 are proteins related to sperm cells and therefore may be reduced or absent in azoospermic seminal fluid samples [14,83,99]. KLK3, TGM4, and SEMG1 are less impacted by lack of sperm cells [84]. Utilization of both a sperm-specific and seminal-specific marker could therefore decrease the chances of false negatives regardless of male fertility.

There are conflicting results about the sensitivity limit of mRNA detection when a seminal fluid sample is diluted [74,76,86]. There is also the possibility of marker erasure when analyzing a fluid mixture [83,95,97]. PRM1 may appear faintly in other fluids, particularly saliva and vaginal fluid, but is not detectable in male urine as it is in the current PSA method [12,69,88]. KLK3 was, however, detected in male urine [88]. The study that sent mock casework to laboratories found that a majority utilized KLK3, PRM1, and SEMG1 [95].

Identification of vaginal secretions has been difficult with current methods due to the complexity and overlap with other fluids [14]. The most researched mRNA markers for vaginal fluid are cytochrome P450 family 2 subfamily B member 7, pseudogene (CYP2B7P1), serine peptidase inhibitor Kazal type 5 (SPINK5), estrogen receptor 1 (ESR1), mucin 4 (MUC4), myozenin 1 (MYOZ1), family with sequence similarity 83 member D (FAM83D), and Dickkopf-related protein 4 (DKK4) [64,65,66,68,70,74,75,76,77,79,80,82,83,85,86,88,89,92,95,98]. Other investigated markers include cytochrome P450 family 2 subfamily A member 6 (CYP2A6), fucosyltransferase 6 (FUT6), serpin family B member 13 (SERPINB13), surfactant associated 2 (SFTA2), glycerol-3-phosphate acyltransferase 3 (GPAT3), and human beta-defensin 1 (HBD1) [66,82,85,89]. CYP2B7P1 is extremely well documented for its high presence in vaginal fluid and its persistence in dilutions and aged samples [65,70,74,76,85,88]. This marker also appears in menstrual blood and rectal mucosa to a high degree [65,74]. In some saliva, blood, and semen samples, CYP2B7P1 has been detected [70,88]. Like with most markers, this is variable per person [70]. SPINK5 is also highly detected and has been shown to be one of the most sensitive markers in vaginal fluid [80,82,88,92]. Two other markers with strong reads are MUC4 and FAM83D [64,68,77,85]. The mRNA marker ESR1 is also found in most vaginal secretions but is less sensitive than CYP2B7P1 and SPINK5 [80,82,88,98]. Unfortunately, SPINK5, MUC4, FAM83D, and ESR1 are often observed in menstrual blood and some saliva samples [76,80,82,85,88,89,92,98]. MUC4 can additionally be detected in rectal mucosa [65].

MYOZ1 is found at lower read counts than the above-listed markers and is not always present in every vaginal fluid sample [86]. While extremely sensitive, HBD1 is one of the least specific mRNA markers for vaginal fluid, as it has also been found in menstrual blood, saliva, and rectal mucosa, [86,87]. GPAT3 was detected in vaginal, menstrual, and blood samples and LCRIS was only found in about half of the vaginal secretions tested [85]. Both DKK4 and CYP2A6 are not strongly found in samples and can vary greatly between persons [64].

Menstrual blood has been a difficult fluid to standardize an identification method for due to it being a combination of cells, blood, and vaginal secretions [14]. The most researched mRNA markers include matrix metalloproteinase-7 (MMP7), matrix metalloproteinase-10 (MMP10), matrix metalloproteinase-11 (MMP11), stanniocalcin 1 (STC1), and left-right determination factor 2 (LEFTY2) [64,65,66,67,70,74,75,76,80,82,83,85,88,95,98]. Other markers are secreted frizzled-related protein 4 (SFRP4), collagen type XII, alpha 1 chain (COL12A1), collagen type VI, alpha 3 chain (COL6A3), matrix metalloproteinase-3 (MMP3), lysophosphatidic acid receptor 3 (LPAR3), and progestagen-associated endometrial protein (PAEP) [64,66,67,82,85,98]. It has been suggested that MMP10 gives one of the highest amounts of reads per sample [64,75]. The day of cycle may influence the expression of this and other markers, however, altering total read counts [67,83,85,99]. STC1 is more sensitive than MMP10, but has been found in vaginal secretions [70,83,85]. Although shown to be quite specific to menstrual blood, MMP11 has less sensitivity than MMP10 and MMP7; this marker may not appear in all menstrual samples [75,76,80,88]. MMP7 has also been shown to strongly appear in menstrual samples; however, this marker has also shown up in rectal mucosa, vaginal fluid, and urine [64,65,67,75,76,80,88,98]. Though it does seem that MMP7 holds up better to dilutions and degradation than MMP11 [76].

Although smaller readings were detected, PAEP showed stronger specificity than MMP7 and LEFTY2 [67]. COL6A3 was found in all tested menstrual samples, but also in numerous peripheral blood samples [85]. LPAR3 appeared in most samples but was also detected in several seminal and vaginal fluids [85]. SFRP4 is virtually undetected in menstrual blood [82]. MMP7, MMP10, and MMP11 were the most popular mRNA markers used during one study about mock casework [95].

Rectal mucosa identification may be helpful in a crime and could be applied to positively identify an object suspected to have been in anal penetration [72]. Suggested mRNA markers include mucin 12 (MUC12), proline, histidine- and glycine-rich 1 (PHGR1), mucin 13 (MUC13), and zymogen granule protein 16 (ZG16) [65,72,74]. Other investigated markers are membrane-spanning 4-domains A12 (MS4A12), chloride channel accessory 1 (CLCA1), meprin A metalloprotease (MEP1A), and caudal-type homeobox 1 (CDX1) [65,72]. Non-mixture rectal mucosa samples have showcased marker reads comparable to vaginal and menstrual secretions, as well as presence of skin and blood markers [98]. MUC12 was found to have good specificity, but low sensitivity in multiple samples [65,74]. MS4A12 demonstrated lower specificity than MUC12 [65]. PHGR1, MUC13, and ZG13 have been suggested due to their resistance to contamination while in mixture, higher sensitivity than other markers, and specificity to rectal mucosa [72].

Although not necessarily a body fluid, skin touch detection has the potential to help pinpoint the origin of a DNA sample or for analysis of complex samples, such as a licked arm [100]. Commonly researched mRNA markers include late cornified envelope 1C (LCE1C), collagen type XVII (COL17A1), C-C motif chemokine ligand 27 (CCL27), and interleukin 37 (IL37) [64,65,66,79,82,95,97,98,100]. Other investigated markers are corneodesmosin (CDSN), filaggrin-2 (FLG2), late cornified envelope protein 2B (LCE2B), skin-specific protein 32 (C1orf68), serpin family A member 12 (SERPINA12), keratin 77 (KRT77), and loricrin (LORICRIN) [64,65,82,95,97,98,100]. LCE1C is the most frequently used marker and often presents with the highest percent read counts in a skin sample [64,95]. COL17A1, CDSN, LORICRIN, and CCL27 have also been readily detected on skin samples [97,98,100]. All mRNA markers listed above have been detected in at least one vaginal, menstrual, rectal, and/or saliva sample [64,95,98]. These markers may also appear on a penile swab when testing for other fluids [79]. It has been suggested that skin samples used in research are not always representative of forensically relevant skin touch samples found at crime scenes, as real evidence may contain very little usable RNA and therefore may cause false negatives with skin mRNA markers [66]. Not all laboratories who implement mRNA markers even test for skin. The study that sent mock casework found that of the few who did, LCE1C was the most common marker, followed by CCL27 and CDSN [95].

Two mRNA markers for nasal secretions have been suggested to differentiate from saliva and other mucosal samples. Opiorphin prepropeptide (OPRPN) and BPI fold containing family A member 1 (BPIFA1) were originally put forth as two potential markers, with BPIFA1 having higher average peak heights [78]. It has been found that BPIFA1 has a strong specificity for nasal secretions; however, the sensitivity is only about 70% [71]. In the mock casework study, only two labs tested for nasal secretions, both using BPIFA1 and STATH [95].

mRNA for urine is not as studied as other body fluids. Despite this, the two markers inositol oxygenase (MIOX) and uromodulin (UMOD), have been found to be specific to urine regardless of sex [80,88].

A prominent goal of recent research is harnessing the ability to target multiple mRNA markers, and therefore fluids, with one run [95]. Multiplexing, or the combination of multiple signals, aims to enhance mixture detection and reduce the number of tests needed for body fluid identification [101]. As noted above, some mRNA markers may appear in another fluid even when the source is not a mixture, creating difficulty during analysis.

When multiplexed, blood mRNA markers may appear in other fluids at lower quantities without necessarily being a mixture, such as a saliva or menstrual stain [74,85,86]. Without being considered a mixture, saliva samples have the potential to contain blood mRNA markers, particularly AMICA1 and CD93 [14,95]. The difficulty with menstrual blood is the potential for overlap with vaginal fluid markers and blood markers; therefore, distinguishing between a menstrual blood sample and a blood/vaginal mixture may be difficult with solely mRNA testing. Blood markers HBB, SLC4A1, and ALAS2 and vaginal fluid markers CYP2B7P1, CYP2BA6, ERS1, and SPINK5 have been shown to not only appear in their respective fluids, but oftentimes in menstrual blood at lower quantities [65,70,74,76,83,85,86,88,95,97,100]. There are menstrual mRNA markers that seem relatively specific, such as PAEP, which could help to indicate if the sample contains menstrual blood, though may not be able to rule out presence of peripheral blood and vaginal secretions if other markers are present [67].

Additionally, due to their compositions, saliva and vaginal secretion markers may appear in their non-respective fluid. One of the most overlapping markers was KRT4, which is an indication of epithelial cells, demonstrating the lack of specificity of this marker for use in forensic body fluid identification [85,86]. The saliva marker HTN3 was found in much lower concentrations in vaginal fluid samples than the vaginal marker FAM83D was found in saliva samples, demonstrating how some markers can be more specific than others [75,85,100].

Some have included mRNA sex markers for increased distinguishability in sample mixtures [65,85,95]. For instance, if the menstrual blood, peripheral blood, and male/female sex markers appear, there is a chance that the sample contains the menstrual blood of a female and the peripheral blood of a male. Examples of sex mRNA markers include R-spondin 1 protein (RPSY1) for males and X-inactive-specific transcript (XIST) for females [65,85,95].

The incorporation of specific genotype testing of mRNA markers has also been performed to increase the ability to identify multiple persons in a sample [64,66,75,82,85,89,98]. This increases the complexity of each test run and the amount of analysis needed. This could be helpful; for example, mRNA testing for semen identification could show that not only is seminal fluid detected, but exclude or include persons based on the genotypes present [98].

There is also the idea of sacrificing sensitivity for specificity. Like most things, mRNA presence can vary per person and is influenced by several factors, including disease state and day of menstrual cycle [14,89]. For standardization to occur across the forensic field, future mRNA investigations will have to determine the most important list of markers to use. Will most panels focus on the more sensitive MMP7 marker or the more specific MMP11 marker for vaginal fluid, or will there always be a need to have multiple markers per body fluid?

One difficulty with mRNA is the instability of the molecule and the likelihood of degradation from improper storage [93]. It has been shown that laboratory-controlled samples maintain mRNA better than samples exposed to environmental conditions, particularly rain [73,102]. Moisture and humidity can be detrimental to the preservation of mRNA, cutting the maximum age of a detectable sample from 71 weeks to a little as 8 weeks [96]. In laundered samples, however, mRNA can remain detectable, especially in cold-washed samples [94]. Not all fluids and markers are equally susceptible to environmental conditions, as saliva and vaginal secretion detection may be reduced before blood and semen detection [96,102,103]. In a study assessing three-year-old samples that had been freeze–thawed several times, semen mRNA markers were detected for all replicates, while vaginal fluid and menstrual blood markers were only partially detected, and blood markers were not detected [76]. It is suggested that sperm-positive seminal fluid performs strongly in mRNA testing of samples due to the sperm cells becoming trapped inside the fabric [104].

**B.**  **miRNA**

MicroRNA (miRNA) are non-coding RNA molecules that are smaller than mRNA, usually around 18–24 nucleotides in length. They play an important role in gene regulation in forensics; miRNA has only been actively investigated for less than two decades, coming onto the scene in 2009 [105].

The most popular technique for miRNA detection in recent research is RT-qPCR. Most studies multiplex known miRNA markers, commonly including at least one for blood, semen, saliva, vaginal fluid, and menstrual blood [13]. Alternatively, the use of RT-PCR followed up with CE was also performed [76]. Recently, high-throughput sequencing and screening of body fluids was carried out to search for new markers, and then these markers were validated using RT-qPCR [106,107].

As forensics continues to expand, the incorporation of new techniques, such as miRNA, requires additional testing to standardize the procedures and markers used. The type of extraction method of a sample used for miRNA processing may impact the RT-qPCR cycle threshold for body fluids differently and may also alter DNA yield. While separate extraction of RNA and DNA could improve yields, it is time consuming and uses additional sample [108]. It has been suggested that coextraction would be preferred and that, after being subjected to DNA extraction, miRNA expression levels are higher than mRNA [109].

As miRNA is still relatively new, current research includes the search for and standardization of reference genes. Previously, small nuclear RNA (snRNA) marker U6 was used as a reference, especially when performing both mRNA and miRNA together; U6 is a conserved component of the spliceosome that is rich in uridine [110]. Several studies have found U6 to be lowly expressed and susceptible to environmental conditions, therefore not a top suitable reference marker candidate [110,111,112]. The marker miR-320a has been proposed as a more stable and highly expressed potential reference [112,113,114]. Other targets, including let-7, miR-484, and miR-191-5p have also been suggested [115,116]. Overall, more work is needed to standardize reference genes for forensic miRNA use before sitewide implementation [116].

While the discovery of microRNA is younger than the first use of mRNA in forensics, there have been promising findings in the application of miRNA for body fluid identification [117]. For blood, miR-451a was introduced as one of the original possible forensic miRNA targets due to its expression in erythrocytes [105,113]. Numerous recent studies have found that not only is miR-451 a highly prevalent in blood, but is also extremely resilient to degradation in laundered and environmentally aged samples [76,85,94,113,118]. Similarly to blood mRNA markers, one difficulty with miR-451a is the presence in both peripheral and menstrual blood [76,112,113,119]. Other explored blood markers include miR16, miR-144-3p, miR-484, miR-182, and miR-21–5p [85,110,112,113].

There has been difficulty with distinguishing saliva and vaginal secretions using miRNA markers [112]. Two of the more frequently used saliva miRNA targets in recent research, miR-205-5p and miR-203-3p, have been found in large quantities in vaginal samples, as well as some semen samples [76,106,112,119,120]. Continual new research in microRNA has given rise to the idea that expression level is a more important factor for body fluid identification than the presence or absence of a marker [106]. For example, one potential saliva marker, miR-223-3p, can be found in other fluids, but is significantly more expressed in saliva and it could therefore be argued to be specific enough for forensic use [106]. This idea could make mixture identification difficult due to overlapping markers, leading to misclassification or the inability to determine mixtures [121].

For seminal fluid detection, the most commonly investigated marker is miR-891a, as some studies suggest the expression of it is significantly higher in semen than other fluids [76,112,115,119]. Other studies disagree, stating that miR-891 is not the most specific or sensitive miRNA for seminal fluid and that this marker can be found at decent levels in feces and other fluids [113,120,121]. Regardless, miR-891a can still be found in seminal fluid stains after laundering, as well as on three-year-old samples [76,94]. Other markers thought to be found more in semen include miR-888-5p, miR-135b-5p, and miR-10b-5p [112,113,116,119,121]. miR-10b-5p read levels were found to not be significantly impacted by environmental conditions such as refrigeration and freezing [118]. Examining markers miR-20b and miR-197 could be used to not only detect the presence of semen, but also to test whether the sample is infertile or fertile [122]. Infertile semen has been found to be more similar to vaginal fluid than fertile semen for the miRNA expression level of miR-197, perhaps showing that there are sperm-specific markers to be found and that greater care is needed for azoospermic samples [122].

As previously mentioned, vaginal secretions have been difficult to distinguish from saliva using miRNAs. Several markers have been investigated for vaginal fluid identification, though one gene has not been solidified as the top candidate. One study determined that the marker miR-372-3p was more specific and sensitive to vaginal fluid than the previously proposed miR-124-3p; however, both appear to have weaker expression values than two other potential markers, miR-193b-3p and miR-1260b [76,112,113,119]. Unfortunately, all miRNA targets investigated for vaginal fluid have been found in other fluids, particularly semen, saliva, and menstrual blood [76,112,113]. It has therefore been suggested that the combination of multiple miRNA markers per fluid would be needed to confidently distinguish between similar fluids, like vaginal secretions and saliva [112].

Menstrual blood and peripheral blood have several overlapping microRNA markers due to their compositions. When investigating potential menstrual blood markers, it has been difficult to not only distinguish from peripheral blood but also from the other common fluids. Suggested miRNAs mi-R412-3p and mi-R185-5p both have been shown to not be specific, as they are expressed in abundance in other fluids, such as semen and saliva [113,116]. A common result is the presence of a marker highly expressed in menstrual secretions, lowly expressed in peripheral blood, but still present in other fluids. There is debate, therefore, on whether these markers could be specific enough to be included in a miRNA panel for body fluid identification. miR-141-3p and miR-200b-3p both appear in all fluids, but expression levels are greater in menstrual than venous blood [112,113,120,121]. Due to overlapping expression levels, there is concern a mixture of peripheral blood and saliva, for example, could incorrectly be labeled as menstrual blood [121]. miR-214-3p has been explored and thought to be more menstrual blood-specific than the other mentions, though this can still be detected in all other fluids at varying levels, with peripheral blood having the lowest expression rate [76,112,116,119].

A ratio of blood miRNA markers miR-451a and miR-21-5p has also been suggested. These two are prevalent in peripheral blood and menstrual blood but not highly expressed in other fluids. The ratio of these marker expression levels has been shown to be statistically significant between the two types of blood, regardless of the age of the individual and day of the menstrual cycle [85]. This offers a unique way to distinguish the fluids and builds on the idea that multiple miRNA markers may be needed for accurate body fluid identification.

For urine and feces, miR-10b-5p was found to be able to separate other fluids from these waste products, though it is still present in semen and vaginal secretions. miR-320c is also abundant in fecal matter [121]. More research is needed for urine and feces identification with miRNA.

Not necessarily a body fluid, but one potential skin marker, miR-3139, has been found by two studies to not be adequately detectable and therefore unsuitable for forensic use [76,112].

Due to length, miRNA is found to be quite stable and resistant to degradation compared to mRNA and is shown to be sensitive enough for forensic use [110,123]. It has even been suggested that miRNA expression values could be more cell type-specific than mRNA [124]. Compared to mRNA, miRNA appears to be more resistant to environmental degradation [73,76]. Light and heat do not seem to impact the detectability of miRNA in general [73,120]. Some markers are more stable than others, possibly due to the GC content of the nucleic acid itself and/or the presence of naturally occurring RNases [111,118]. Freezer conditions of −20 °C also do not negatively affect miRNA detectability, though refrigeration at 4 °C has conflicting results [111,118]. Like mRNA, miRNA is very susceptible to damage from rain and humidity [73,111]. It is important to note that expression levels of microRNA can also be altered by disease and miRNA is easily transferred in laundered samples [94,105]. As discussed, mixtures may be particularly difficult with miRNA due to many markers being present at some level in all fluids.

**C.**  **EPIGENETICS**

Epigenetics is the study of outside influence on gene regulation, with one of the most common areas of study being DNA methylation. Epigenetics is an active method of research in forensics, though it is more popular in other areas, such as age estimation, than in body fluid identification [125]. DNA methylation is the addition of a methyl group to a nucleotide to regulate the gene’s expression. The idea for epigenetic application in forensics is that tissues are regulated differently and therefore will have different methylation patterns, allowing for body fluid origin classification [126]. Oftentimes, CpG sites are investigated, which are areas in the DNA where a cytosine is followed by a guanine, allowing for that cytosine to be open to methylation. Some methylated loci are associated with known genes while others are not [127]. It is important to note that a locus with no methylation in one fluid compared to others could also be a potential marker for discrimination [128].

In order to analyze methylation with certain protocols, the methyl groups need to be secured, and this is usually performed with bisulfite conversion. This changes unmethylated cytosines to uracil, which will become thymine after PCR amplification; the methylated cytosines will remain as cytosines [127]. Once the CpG sites are secured, detection of methylation patterns can be carried out with a variety of techniques. One of the most common methods for forensic epigenetic research is the SNaPshot assay, which is a fluorescent labeling technique often used for single nucleotide polymorphisms (SNPs), but can be adapted for methylation use. SNaPshot is followed by capillary electrophoresis (CE) for analysis where fluorescent peaks can be used to assign the presence of methylated cytosines to a sample. This technique is easy to integrate into forensic workflows as CE is used by many for DNA analysis. A comparison of multiple laboratories showed that the use of different extraction methods and genetic analyzers did not have a significant impact on the outcome of the SNaPshot assay [129]. Methylation-sensitive restriction enzyme (MSRE) use in PCR or RPA followed by CE has also been employed, as this method cannot cleave methylated sites, does not require bisulfite conversion, and utilizes equipment already available in crime labs [125].

Pyrosequencing is the gold standard technique and is based on the release of light; it has been used in previous epigenetic forensic work, though is less common in current body fluid identification epigenetics research [126,127]. High-resolution melting analysis (HRM) can also be performed to identify methylation markers by comparing melt curves of DNA strands to determine the nucleic acid present. One study found pyrosequencing to have comparable results with HRM [130].

Next-generation sequencing methods potentially overcome previous problems but still require reproducibility testing for forensic epigenetic use. Massively parallel sequencing (MPS) has the ability to process a large number of samples simultaneously and with high accuracy [131]. Studies have found MPS to work effectively for epigenetic body fluid identification and state that MPS is comparable to SNaPshot results [128,131,132]. Nanopore sequencing has also been investigated and does not require bisulfite conversion for methylation detection [133,134].

Since body fluid identification using epigenetics is still rather new, the fluid-specific markers have not been standardized yet. Two human blood markers, cg06379435 and cg08792630, have been investigated recently in several studies and found to be robust enough for possible forensic use [129,131,133,135,136]. One study was able to corroborate enzymatic presumptive positives for blood from previously frozen forensic evidence from cases in 2006, 2009, and 2016 [137]. Other new studies have focused on finding novel potential CpG sites or further investigation into markers suggested by other authors [127,130,135,136,138]. The potential marker cg04011671 was found to be easily detected in venous blood samples but also gave a positive result for menstrual blood [139]. Notable mentioned markers include cg25922751, cg24301930, cg00438740, and cg13763232 [127,130,138].

For saliva, one locus investigated, cg09652652, is associated with the family with sequence similarity 43 member A (*FAM43A*) gene. This marker has been found in saliva and saliva-related samples, including lip, tongue, and nasal swabs, as well as in chewing gum and cigarette butts, with minimal methylation expression in other fluids, semen and blood especially [140]. This marker was also used on old forensic casework samples and produced positive saliva methyl profiles, which agreed with enzymatic testing [137]. Some studies have focused on a more saliva-specific method by pairing marker cg21307118 or cg26107890 with SNPs [132,136]. There can still be difficulty with distinguishing saliva and vaginal fluid, particularly as a mixture [127,131,132].

Two CpG sites, cg03902386 and cg03282313, could potentially be used for both saliva and semen, as these two are virtually always methylated in semen and rarely in saliva [138]. The locus cg17610929 is associated with the acid-sensing ion channel 4 (*ASIC4*) gene and cg26763284 with the plectin (*PLEC*) gene [129]. Both have been used in several studies across laboratories with promising results at semen determination [129,131]. A method for semen and donor identification takes the combination of cg26763284 and a Y chromosome marker, allowing for more information to be obtained from a single test; this method is currently only for semen with sperm present [141,142,143]. Examples of other potential semen-specific markers include semen cg20864568, cg09245584, and cg13885748 [127,128,136]. Work is still needed to determine if the markers listed above can be regularly applied to azoospermic seminal fluid or if additional methods are required.

DNA methylation loci for vaginal fluid identification research have primarily revolved around cg09765089-231d and cg26079753, the latter of which is associated with the *HOX* transcript antisense RNA (*HOTAIR*) gene [129]. There has been overlap found for cg26079753 in urine and skin, as well as very low levels in menstrual blood [135,144]. This marker was still able to be clearly detected in various semen and vaginal fluid mixtures [135]. The methylation expression level was significantly different for cg26079753 between pre- and post-menopausal samples [127]. Although current enzymatic testing may be negative for vaginal fluid, these two markers positively identified vaginal fluid on old forensic evidence, allowing the STR profile found to be linked to a specific body fluid [137]. Other potential vaginal fluid markers include cg08751438, cg05558714, cg07452397, and cg01774894 [130,136,138].

CpG loci cg18069290 (associated with solute carrier family 26-member 10 (SLC26A10) gene) and cg09696411 have been researched for use in menstrual blood identification [129,131,135,137,144]. The novel markers cg04255276 and cg24772753 have also been suggested for menstrual fluid [130,138]. For menstrual blood, there can be overlap of methylation expression of these three markers not only with vaginal fluid, but also venous blood [131,138,139]. Additionally, it has been found that methyl levels can vary in menstrual blood based on the day of a person’s cycle [144]. Although there have been promising results from these investigations, further research is needed to eliminate incorrect labeling and improve mixture analysis.

DNA methylation is known to be impacted by a variety of factors, including disease, age, ethnicity, and lifestyle [125]. Therefore, methylation levels can vary between individuals, and some markers may not be consistent enough for forensic use [144]. Not only this, but marker and sample type can influence how degraded DNA methylation may become from storage conditions [145]. Previous studies found difficulty with fluid-specific markers, but suggest that expression levels are more relevant for distinguishing than marker presence, similar to what was previously mentioned about miRNA [146]. Also, it is suggested is that too many markers in one panel may be disruptive to testing [144].

Though bisulfite conversion is often necessary to solidify the methylated areas, the DNA is altered and possibly unable to be used for other purposes [125]. This may be a problem when there is limited sample available for testing. As mentioned, some protocols investigated have combined SNP or STR markers with CpG sites in order to acquire more information from a single sample [132,142,143]. The use of newer techniques that do not require bisulfite conversion with a combination of marker types could provide extensive information [125,133,134].

As of now, the threshold levels for CE of methylated markers are not standardized across laboratories, which could influence the labeling of a sample as positive or negative [129].

In forensics, there is little current research focused on degraded or environmentally impacted sample testing for body fluid identification through epigenetics. Mock forensic casework samples, such as mixtures on swabs, cigarette butts, and tissues, have been included [140,143,144]. Aging in a lab setting has also been performed, which shows no significant reduction in the ability to detect methylation markers [136,147]. Real forensic casework samples were re-examined with epigenetic protocol and found to corroborate previous findings and reveal new information, like the fluid origin of the DNA present [137]. Thermal degradation at 95 °C ranging from 15 min to 8 h showed that heat significantly impacted marker detection with capillary electrophoresis, though not all markers were affected equally [136]. Clinical studies on CpG sites reveal that short-term (a few days) storage in refrigeration may not alter methylation results, but long-term freezing decreases detection of methylation levels [145,148].

**D.**  **MICROBES**

Microbial forensics has historically revolved around bioterrorism and biological agents, starting with the anthrax letter attacks of 2001 in the U.S. [149]. Microbes have been investigated in other areas of forensics, including cases of death by drowning and post-mortem interval determination [150]. For body fluid identification, there is an abundance of clinical information to pull from, as human microbiomes are very well documented for medical purposes. Most of the recent microbial forensic research for body fluids is about environmental conditions, forensic circumstances, and the ability to distinguish fluids after exposure. It is known that microbiomes can be altered by age, disease, ethnicity, location, diet, and habits. Menstrual blood bacteria have also been shown to fluctuate during a person’s cycle [151].

In order to determine the abundance of bacterial species present, the bacteria 16S ribosomal RNA gene (16S rRNA) is targeted. This gene contains conserved and hypervariable regions. Utilization of only the V3 and V4 regions is popular because the target length is shortened without compromising efficacy [152,153,154,155]. These two regions have been shown to provide comparable results to full length reads; however, use of the full gene length could provide more accurate and specific taxonomy [156,157]. Most new research sequences all bacteria present in a sample [152,153,154,155,156,157,158,159,160,161]. Another method is to target specific bacteria species and proceed with capillary electrophoresis [162,163].

Human peripheral blood is essentially sterile, and bacteria found in a blood sample is often linked to infection or contamination [161].

The semen microbiome appears to be more diverse and varies more per individual than the vaginal microbiome [155]. The main bacterial genus used for semen identification is *Corynebacterium* [154,159,164]. Other prominent genera include *Prevotella*, *Staphylococcus*, and *Pseudomonas* [154,155,157,159]. These bacteria are also found on human skin around the body and on the penis [160,162]. While there is growing work into categorizing the semen microbiome, more research is needed to compete with current identification methods.

Saliva is known to contain bacteria as part of a healthy microbiome [151]. The *Streptococcus* genus is commonly found in the oral cavity [149]. Specific species of *Streptococcus* used in recent forensic body fluid research include *S. salivarius*, *S. neisseria*, and *S. oralis* [157,158,162,164]. The saliva microbiome is shown to be distinguishable from vaginal, semen, and skin samples due to the abundance of oral-specific bacteria [158]. The oral microbiome is subject to alterations by diet, oral hygiene habits, smoking, and geographic regions [158,165,166]. Distinguishing saliva from nasal secretions has also been investigated with promising results by using *Staphylococcus*, as this genus is rarely found in saliva [153].

The vaginal microbiome tends to be dominated by a select group of bacteria, mostly belonging to the *Lactobacillus* genus. There is some variation between geographic regions, but most individuals have an abundance of *Lactobacillus*. *L. crispatus*, and *L. iners,* which are extremely common and are shown to be vaginal-specific species [151]. *L. gasseri* and *L. iners* have also been identified as vaginal bacteria [154,163]. Other common vaginal bacteria genera include *Gardenerella* and *Bifidobacterium* [152,163,164]. Several *Lactobacillus* species are known to be human-specific vaginal bacteria, including *L. crispatus* and *L. iners* [163]. The vaginal microbiome has been shown to be used to successfully distinguish vaginal fluid from other fluids [152,159,163]. Menstrual blood and female urine are often too similar to vaginal fluid to rely solely on microbial analysis for identification [159,163]. Additionally, different areas of the female genitalia can have different levels of bacteria and there is also microbial exchange during sexual intercourse, which are two factors immediately relevant for forensic sampling [160]. The vaginal microbiome can be influenced by other factors, including infection and antibiotic use [151,163]. Individuals with health complications, such as cervical erosion and vaginitis, are still shown to have predominantly *Lactobacillus* bacteria. Age, however, seems to be an incredibly important factor for vaginal microbiome contents, with post-menopausal samples being significantly more difficult to successfully positively identify [163]. Due to these variables, it is important to note that relying on one or two species for vaginal fluid identification may result in increased false negatives.

As previously mentioned, the bacteria present in menstrual blood is extremely similar to vaginal fluid and the two cannot be distinguished using microbial forensics at this time [159,163].

The skin contains a variety of bacteria across the body that can change in abundance based on hygiene, geographic location, temperature, and moisture [162,167]. *Staphylococcus* is a known skin bacterial genus that could be used for skin identification, though it is commonly associated with disease [167]. Alternatively, the *Corynebacterium* and *Cutibacterium* genera have also been suggested for use [158,162,167]. The *Corynebacterium* genus is also found in semen and in male genitalia, as previously mentioned [154,160]. *Cutibacterium acnes* is a species specifically linked to acne and can be found around the body, making it a potential skin-specific microbe for forensic use [162,167].

Urine varies greatly between sexes. As discussed, the bacteria in female urine are very similar to vaginal fluid and thus distinguishing the two based on microbes is not currently doable [151,159,163]. Male urine is understudied in recent research, though *Streptococcus* has been suggested as a potential microbe [159]. This genus is often found in abundance in saliva, however [151].

Fecal matter is known to have an exorbitant number of microbes compared to other body sites, with species variability caused by factors such as diet, genetics, lifestyle, and disease. The two dominant phyla are Bacteroidota and Bacillota, which include common gut genera such as *Prevotella*, *Clostridium*, and *Faecalibacterium* [167]. One of the most dominant species is *Faecalbacterium prausnitzii*, which has been suggested for fecal matter identification [162]. Much more research is needed for microbes in fecal matter in a forensic context.

Since bacteria are living organisms, it is important to understand how environmental conditions may alter microbial content. Sterile swabs kept at room temperature indoors, up to 65 days, tended to have no significant difference in microbial diversity from fresh swabs [152,154,161,164]. There is potential for bacterial growth in warm conditions, so storage at lower temperatures is recommended [152].

Microbes are everywhere and the surface a fluid is found on could impact the microbial reading. Environmental bacteria can increase on exposed samples over time, especially those not on sterile surfaces [155,157,161]. Mock samples deposited on a bedspread were seen to have environmental bacteria in microbial analysis after 7 days of room temperature exposure; fluids deposited on the floor showed significant contamination from environmental bacteria [161]. It is suggested from these findings that deposition location could have a more drastic effect on the microbial content of a fluid sample than exposure [161]. Semen in soil and semen in an enclosed plastic bag for 15 days showed a decrease in microbial diversity compared to fresh samples and semen deposited on a variety of fabrics for 15 days [155]. *Lactobacillus* in vaginal fluid and menstrual blood continuously showed durability to time and location exposure [157,161,163]. Microbial content of vaginal fluid also showed stability against high concentrations of added inhibitors, such as 125 ng/μL of humic acid, with core bacteria species remaining detectable. Aged real forensic casework samples up to 5 years old still had detectable vaginal bacteria present [163].

Of all relevant body fluids, vaginal fluid has the highest potential for microbial forensic use due to the specificity and abundance of bacteria in the vaginal microbiome. However, there is overlap with menstrual blood and female urine, but a combination of techniques could be used. For instance, the detection of sexual assault samples that include seminal fluid and vaginal fluid could benefit from microbial testing and other semen detection methods to obtain the clearest picture [152].

**E.**  **OTHER RESEARCH**

Other techniques for body fluid identification have been investigated to a lesser extent in current research.

Proteomics is the analysis of proteins, which are the products of genes. In addition to body fluids, proteomics is utilized in other areas of forensics, including post-mortem interval and time deposition [168]. Some proteins, like semenogelin, have already been integrated into forensic serology [49]. The mRNA expression of this protein has also been investigated [14]. Other notable proteins researched in proteomics that have been mentioned above in other techniques include PSA, statherin, HBB, MMP10, and alpha amylase [5,41,69,169,170]. It is known, however, that the protein form of a gene is more stable than the mRNA form [170]. Protein analysis is performed using mass spectrometry (MS), which is predominantly used in forensic toxicology for drug analysis [169,171]. Therefore, the equipment for proteomic work may already be available for use in a crime lab. In fact, the combination of protein analysis with biological and toxicological areas of forensics has been suggested as a means of optimizing equipment and sample use, as well as potential to detail a fuller story of the crime [168]. The addition of machine learning in proteomics has also been explored for the determination of the best method approach and likely identification of a fluid, though more research is needed [172]. Regardless, the ability to perform targeted and untargeted liquid chromatography MS (LC-MS) expands the possibilities of proteomic use for stains with no presumptive origin determined [168].

MS analysis of seminal fluid for the semenogelin protein found high sensitivity down to 0.0001 μL of semen and the protein was able to be detected in mixtures with vaginal fluid, saliva, and menstrual blood [173]. For menstrual blood, proteomics has similar difficulties as other methods with similarity to venous blood and vaginal fluid, as well as variability given the day of the cycle [170,174,175]. However, there are certain proteins found in menstrual blood but not venous blood, and vice versa [175]. Exclusion instead of the conclusion of fluid origin may, therefore, be more probable in these cases [172]. Further clinical understanding of menstruation and menstrual blood would benefit forensic body fluid identification and should be supported by the forensic community [174]. Recent research focused on saliva identification using statherin with 71–92% positive identification depending on processing methods [176].

One concern with proteomics is integration into the current workflow. Post-DNA extraction waste products of blood, semen, and saliva were used for MS analysis of peptides and showed that important body fluid proteins, like semenogelin, could still be detected [177]. This finding demonstrates that proteomics could be added to the investigation without negatively impacting DNA analysis. An investigation into several fluids found that there are possible proteins with higher discriminatory power than popular ones and that reliance on a single protein may result in missed findings [178]. As with other techniques mentioned above, there can be overlap between fluids and therefore the ratio of protein abundance may be an important tell for fluid origin [170,177]. With further research, proteomic methods can become faster and more automated for forensic use [173]. Overall, more research is needed for proteomics used in forensics.

Non-invasive methods of detection have also been researched. One advantage of the following methods is the ability to detect without destruction of the sample, which allows for further identification of the sample analyzed. Utilizing specific wavelengths to illuminate the natural fluorescence of proteins in body fluid could provide an efficient screening method to categorize a stain on-site. It is suggested that 425–450 nm is specific for vaginal fluid, but more research is needed for this and all fluids [54,179]. Raman spectroscopy uses the scattering of light by vibrating molecules to differentiate substances and is a rapid system currently used for drug analysis [180]. It is possible that the Raman spectra for body fluids could be used for body fluid identification, or even for further information, such as ancestry [180,181]. There is the possibility of using Surface-Enhanced Raman Spectroscopy (SERS) for distinguishing between menstrual and venous blood due to a unique excitation of menstrual blood, though this still needs to be compared with other body fluids [182]. SERS has also shown to be able to differentiate between human and non-human blood [182]. Recent research into near-infrared (NIR) Raman spectroscopy has strong potential for blood stain identification, though is less potent for saliva and semen stains [183]. An important factor of Raman spectroscopy for fluid stains is the presence of different fabric types and dyes. Advanced research on fabric types and colors would be beneficial to building a robust Raman tool for body fluid identification for casework samples [182,184,185]. The incorporation of chemometrics, or machine learning, could increase the speed and accuracy of such tool development due to the high amount of pattern recognition data needed [185].

Attenuated total reflection–Fourier-transform infrared spectroscopy (ATR FT-IR) is another form of non-destructive spectroscopy, which produces visual spectra for analysis. FT-IR has been used in forensics for fingerprint analysis and post-mortem interval [186,187]. The visual spectra provided by body fluids, such as semen and vaginal fluid, can look strikingly similar but can be distinguished using mathematical models [188,189,190]. Mixtures may be more difficult to deconvolute, as well as menstrual and peripheral blood [188]. The data analysis and mathematical portion of FT-IR for body fluids have not been standardized, and it is suggested that models may vary slightly based on the substrate [189]. The use of ATR FT-IR for body fluid identification is still relatively new and requires more research before being applied in regular forensic use.

## 4. Discussion

While the field of forensic science has come a long way over the years, there is always a desire to improve the sample analysis workflow, particularly when handling small amounts of evidence. There are a whole host of new technologies and new clinical findings that could be applied in a forensic context. Currently, there is a need for increased speed and precision for crime scene evidence processing. This is already being explored for rapid DNA analysis of samples in the lab or at a crime scene [191]. The drive to clear up the backlog of unsolved cases while also handling new cases swiftly is sometimes at odds with the unfortunate reality of cost efficiency, disproportionally impacting sexual assault cases [192,193]. Efficient and informative methods for body fluid identification should be prioritized to fill knowledge gaps that can then be integrated into real casework.

One of the most difficult body fluids to develop an identification method for has been vaginal fluid due to similarities with menstrual blood, semen, and saliva. Vaginal secretions do not currently have a standardized identification protocol [14]. It could be suggested that since there are presumptive and/or confirmatory tests for peripheral blood, semen, saliva, urine, and even menstrual blood, the lack of a vaginal fluid test is a pressing matter in forensic serology. With the goal of efficiency in mind, novel vaginal fluid identification protocols that can include other fluids or DNA processing should be prioritized.

Each molecular biology technique mentioned in this review has advantages and disadvantages for forensic analysis (Table 2). mRNA has been a thoroughly studied potential route for body fluid identification purposes. mRNA can be coextracted with DNA so that both nucleic acids can be analyzed from a single sample extraction [76]. Specific mRNA markers can be targeted and detected using capillary electrophoresis, a piece of equipment already used in forensic laboratories [88]. Sequencing of mRNA has the potential for observation of a wider variety of markers and for donor mixture distinguishment using SNPs and sex markers [64,85,89,98]. Massive Parallel Sequencing is not yet commonplace in forensic workflows, and the incorporation of sequencing can be expensive and time consuming [75]. There is also the problem of mRNA instability from environmental exposure [73].

miRNA, however, is more resistant to degradation compared to mRNA, although both are susceptible to humidity and rain [73,110]. Current research on miRNA suggests that expression levels of markers may be more important than presence, which could be an issue for mixture identification [113]. Multiple miRNA markers for each fluid could thus be utilized to help clarify findings; however, this could still be insufficient [112]. Capillary electrophoresis is common with miRNA, so some studies have elected to combine mRNA and miRNA for body fluid identification. By doing so, a common piece of equipment is being utilized, and a range of information is being generated [73,76]. RT-qPCR of both mRNA and miRNA has also been performed [94,113]. The two types of RNA can be coextracted, which simplifies part of the process of combination [76]. By combining two types of nucleic acid, the hope is to offset the limitations from each other.

Similarly to miRNA, methylation patterns in epigenetic analysis are suggested to be more relevant than marker presence [146]. The protocols for extraction and stabilization of methyl groups have previously included bisulfite conversion, which affects the DNA, is time consuming, and an additional expense [138]. New research has developed MSRE in PCR to bypass this conversion step and quicken the process while also allowing easier integration into the current workflow [125]. However, the lack of standardized methodology, despite having the equipment necessary, could be a handicap for the implementation of this technique in crime labs. One difficulty with epigenetics is the names of the markers. They are not as easy to remember and keep straight as mRNA markers as they are not always associated with known genes [127]. A personal recommendation for the establishment of epigenetics in body fluid identification is the addition of colloquial names for common markers once they are established.

The technique that appears to be most specific for vaginal fluid is the analysis of bacteria present, as the human microbiomes are well documented clinically [151]. Microbial analysis could also be important for saliva and fecal matter identification [151,162]. Not all body fluids could be accurately detected with just microbes. Peripheral blood is virtually sterile, and seminal fluid is often extremely diverse in bacteria species [155,161]. It is possible that current methods for identification of blood and semen could be more powerful than the use of bacteria. Vaginal fluid also cannot be distinguished effectively from menstrual blood or female urine [163]. Since the target for microbial analysis is the 16S rRNA gene, coextraction with DNA and/or other RNA can be performed, creating the potential for combined techniques. For instance, the combination of miRNA for semen and 16S rRNA for vaginal bacteria has been suggested to overcome difficulties regarding vaginal fluid identification and mixture analysis [121]. Some techniques elect to sequence all bacteria in a sample, acquiring immense information and reducing the problem of variability of dominant bacteria between people [160]. CE of specific species is also an option, though this requires deliberate species selection [163]. There is also the problem of environmental bacteria contamination. Although sample microbes can be stable over time, naturally occurring bacteria can assimilate into the sample [161]. Microbial exchange between persons, such as during sexual intercourse, could be harmful or helpful depending on the scenario [160]. Microbial forensics has potential in body fluid identification and has been explored in other aspects of forensics, such as personal identification, drowning cases, and post-mortem interval [150,167].

Proteomics may be difficult to integrate into current forensic DNA workflows because it utilizes MS, which is not common in a forensic biology lab. However, MS is widely used in forensic toxicology and forensic chemistry labs; so, if the DNA analyst has access to these laboratories, it would be possible to carry out. Though, it would depend on the jurisdiction and capabilities of the crime lab. Proteomics essentially analyzes the protein component of the genes targeted in body fluid mRNA analysis, which could be beneficial if the mRNA is severely degraded [169]. Overall, proteomics for body fluid identification is understudied and requires more research.

Non-destructive methods could potentially be an important first step in body fluid analysis. Raman spectroscopy is already used in the field for drug analysis, so an on-site non-destructive body fluid identifier could be very useful, even just for presumptive testing [180]. FT-IR is also used in other aspects of forensics [189]. These two methods are in their infancy for body fluid identification. More research and math model development are needed before implementation, but non-destructive analysis is an important proposal when the evidence sample is small.

## 5. Conclusions

**Figure 1:**
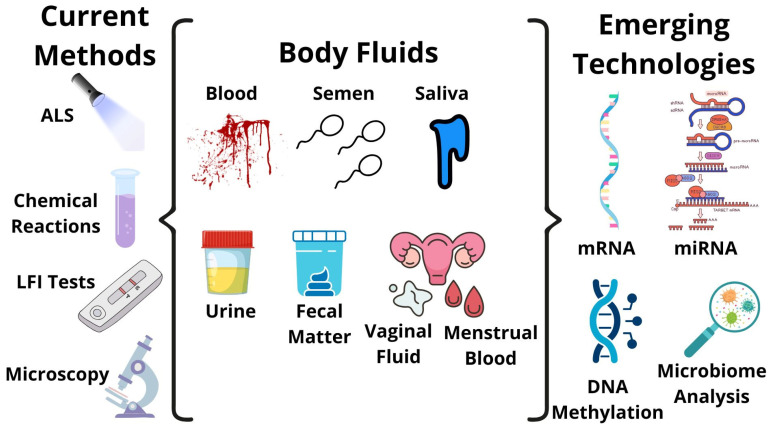
Summary figure of the current methods (**left**) and emerging techniques (**right**) described in this review.

The current research into molecular techniques for forensic body fluid identification shows exciting promise for further development of the field (Figure 1). Each area has its own advantages and disadvantages (Table 2). Before the community decides on the widespread implementation of any method mentioned here, there is a need for consensus on the most important aspects of new techniques, whether that be specificity, sensitivity, or more intricate analysis to have the best of both. The reality of cost and efficiency can also not be forgotten when proposing methods for integration; not every crime lab has the latest sequencer or automated extraction equipment. Future research should prioritize methods that can be integrated without detracting from current workflows or creating excessive new costs. Moreover, any new method, before being applied to a forensic case, should be subjected to internal and inter-lab validation, both with mock and case samples, including degraded and mixture samples, to ensure the validity of the technique in different scenarios. Additionally, if the lab is accredited and follows ISO/IEC 17025, the new method should follow the validation expectations as well as current OSAC (The Organization of Scientific Area Committees for Forensic Science) guidelines.

mRNA use is the most active research subset currently and has a long history to prove its potential place in body fluid identification. Already, there have been inter-lab investigations, environmental degradation analysis, proposal of panels, and analysis of complex fluid scenarios [64,67,73,86,95]. mRNA seems the most likely candidate for future method adoption, regardless of any disadvantages. But do not count out the other techniques mentioned just yet. Combination methods could acquire a great deal of information from each sample and simplify future analysis. More forensic research and novel clinical findings will allow body fluid identification to become even more refined in the near future. Going forward, validation and casework studies are essential for proving the reliability of any new method.

## Figures and Tables

**Table 1:** Summary of the current methods applied by crime labs.

Body Fluid	Chemical Techniques	Immunochromatographic Tests	Microscopy
Blood	Phenolphthalein Leuco-malachite Luminol	Heme Glycophorin	Takayama
Saliva	Amylase	Amylase	—
Semen	Acid phosphatase Prostate-specific antigen	Prostate-specific antigen Semenogelin	Sperm identification
Vaginal Fluid	Acid phosphatase	—	Dane’s staining method Microbes
Menstrual Blood	—	D-dimer	—
Urine	DMAC assay Creatinine	Tamm–Horsfall protein	—
Feces	—	—	Microbes

**Table 2:** Summary table of the advantages and disadvantages of emerging techniques for body fluid identification.

Technique	Advantages	Disadvantages
**mRNA**	Coextraction with DNA Combining marker types Uses capillary electrophoresis, RT-qPCR, or sequencing	Sensitive to environmental conditions Markers overlap between fluids
**miRNA**	Coextraction with mRNA Resilient to environmental conditions Combining marker types Uses capillary electrophoresis, RT-qPCR, or sequencing	Reference genes not yet standardized Markers overlap between fluids Difficulty with mixtures
**Epigenetics**	Combining marker types Uses capillary electrophoresis, HRM, MSRE, or MPS	Bisulfite conversion damages DNA Pyrosequencing, the gold standard technique, is not available in crime labs
**Microbes**	Clinically well documented human microbiomes Species specific	Not all fluids are rich in microbes
**Proteomics**	Uses mass spectrometry Proteins generally stable	Markers overlap between fluids
**Spectra**	Non-invasive	Understudied

## Data Availability

No new data were created or analyzed in this study.
